# Assay for screening for six antimalarial drugs and one metabolite using dried blood spot sampling, sequential extraction and ion-trap detection

**DOI:** 10.4155/bio.10.147

**Published:** 2010-10-20

**Authors:** Daniel Blessborn, Susanne Romsing, Yngve Bergqvist, Niklas Lindegardh

**Affiliations:** 1 Dalarna University College, Borlange, Sweden; 2 Department of Physical & Analytical Chemistry, Uppsala University, Uppsala, Sweden; 3 Faculty of Tropical Medicine, Mahidol University, Bangkok, Thailand; 4 Center for Clinical Research Dalarna, Falun, Sweden; 5 Nuffield Department of Clinical Medicine, Centre for Tropical Medicine, University of Oxford, Oxford, UK; Tel.: +66 2203 6368; Fax: +66 2354 6018; danielb@tropmedres.ac

## Abstract

**Background:**

More parasites are becoming resistant to antimalarial drugs, and in many areas a change in first-line drug treatment is necessary. The aim of the developed assay is to help determine drug use in these areas and also to be a complement to interviewing patients, which will increase reliability of surveys.

**Results:**

This assay detects quinine, mefloquine, sulfadoxine, pyrimethamine, lumefantrine, chloroquine and its metabolite desethylchloroquine in a 100-µl dried blood spot. Most of the drugs also have long half-lives that make them detectable at least 7 days after administration. The drugs are extracted from the dried blood spot with sequential extraction (due to the big differences in physicochemical properties), solid-phase extraction is used as sample clean-up and separation is performed with gradient-LC with MS ion-trap detection.

**Conclusion:**

Detection limits (S/N > 5:1) at 50 ng/ml or better were achieved for all drugs except lumefantrine (200 ng/ml), and thus can be used to determine patient compliance. A major advantage of using the ion-trap MS it that it will be possible to go back into the data and look for other drugs as needed.

**Figure 1.**
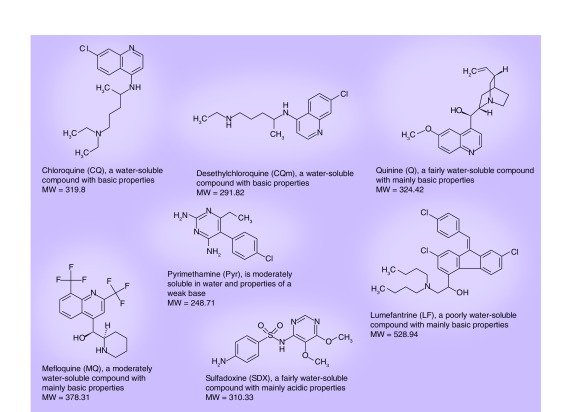
Antimalarial drugs and one metabolite used in this screening assay.

**Figure 2.**
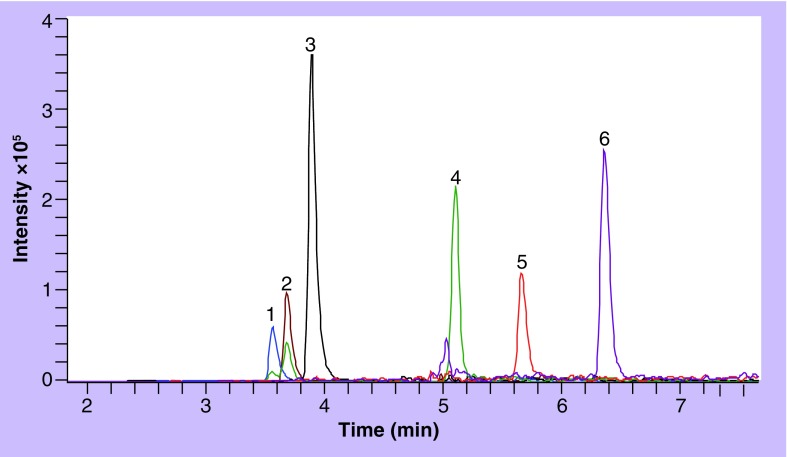
Extracted ion chromatogram of each drug in overlay mode from the lowest quality control sample (250 ng/ml blood spot). Elution order: 1: desethylchloroquine; 2: chloroquine; 3: quinine; 4: pyrimethamine; 5: sulfadoxine; 6: mefloquine hydrochloride.

**Figure 3.**
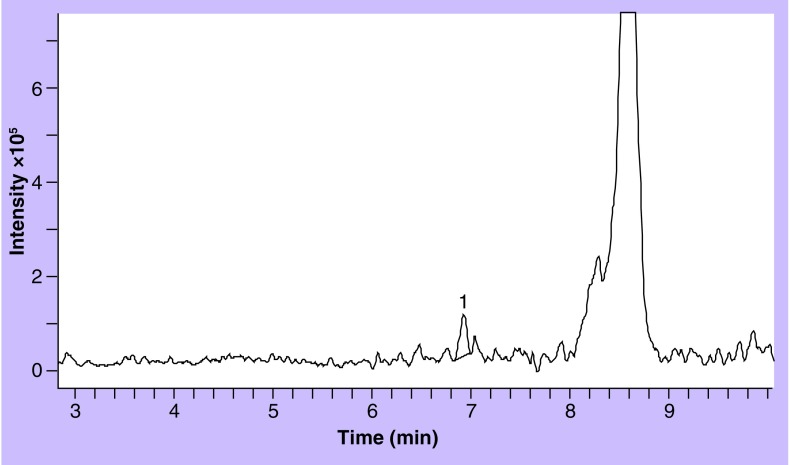
Extracted ion chromatogram of lumefantrine (peak 1) from the lowest quality control sample (250 ng/ml blood spot).

Malaria is an infection caused by the one-celled parasite *Plasmodium*, and the most severe type of malaria is caused by *Plasmodium falciparum*. Malaria parasites are transmitted by the female mosquito of the genus *Anopheles* when it injects its saliva into the human body [1]. Malaria is present in over 100 countries in the world with an estimated 250 million malaria cases a year causing nearly a million deaths, mainly in children under 5 years of age [2].

There are several drugs against malaria, but unfortunately many of these are starting to lose their effect due to development of drug resistance by the parasite. The long use of drugs as monotheraphy is one of the main reasons, but lack of compliance and counterfeit drugs with too low or no drug content are certainly contributing factors [3].

Therapeutic drug monitoring (TDM) is an important part in monitoring the correct drug levels. If drug levels are too low it could increase the chance of emerging drug resistance. The main constraints for successful TDM in the field are poor facilities at the study site in combination with storage and transportation issues. Most biological samples have to be kept refrigerated or frozen and disrupting the cooling chain may result in drug degradation [4]. One sampling technique that can overcome these problems is the dried blood spot (DBS) technique. The technique is less invasive than venipuncture and is more cost effective for storage and transportation. DBS samples generally increase stability of the drug compounds and also reduce transmission of infectious disease (e.g., HIV) making them safer to handle for laboratory personnel [5]. The drawback with this method is that the analysis of drug concentrations can often be quite complicated and tedious. Introduction of the paper matrix makes it more difficult to extract the drug from blood with high recovery. The sample volume is also small (i.e., typically ∼100 µl) making it more difficult to achieve adequate sensitivity for drugs with low therapeutic concentrations in blood. Some methods have used 200 µl of capillary blood, although more than 100 µl is difficult to collect, especially in small children.

Determining antimalarial drug pressure in an area usually includes interviewing patients. However, this might not give an accurate and complete picture of drug use in that area. In a short report from 2002, Legros *et al.* conducted a study on clinical efficacy of chloroquine (CQ) and sulfadoxine (SDX)-pyrimethamine (SP) in children. In this study, urine testing was carried out on 53 children. Of these, 34 were positive for CQ or SP and only 17 (50%) of the mothers remembered whether their children had been given antimalarial drugs prior to enrolment [6]. Hildenwall *et al.* carried out a study comparing caretakers’ reports of drug intake of CQ and SDX with drug levels in blood. The study showed that many caretakers were unaware of what drug had been given to the child and under-reporting drug intake was common. They concluded that caretakers’ reports of drug intake have limited validity [7]. Studies from Tanzania and Malawi have also shown poor correlation between caretakers’ stated intake of antimalarial drugs for their child with measured drug levels in blood or urine [8,9].

The aim of this qualitative method is to help determine drug use in areas where over-the-counter sales and self-medication are common. It can also to be used as a complement to interviewing patients that will increase reliability of the survey. It will be especially beneficial to deploy where a change in official treatment recommendation has recently taken place with the main objective to detect some of the most common antimalarial drugs often used in self-medication, for example CQ (and its metabolite), quinine (Q), SDX, pyrimethamine (Pyr), but also lumefantrine (LF) and mefloquine. There is also a metabolite to mefloquine, but it was excluded from this method since it was the only drug that required the LC–MS to work in negative mode and this would leave a 1–2 min gap where no other drugs could be detected.

## Experimental

### ▪ Chemicals & materials

The Pyr and Q were obtained from Sigma (MO, USA). Mefloquine hydrochloride (MQ) was obtained from Hoffman-LaRoche (Basel, Switzerland). CQ, desethylchloroquine (CQm) and SDX were obtained from LGC Promochem (Teddington, UK). LF was obtained from Novartis Pharma AG (Basel, Switzerland). Acetonitrile, methanol and water (LC–MS grade) were obtained from JT Baker (MT, USA). Formic acid and ammonium formiate (LC–MS grade) were obtained from FLUKA (Sigma-Aldrich). Acetic acid (HAc) and hydrochloric acid (analytical grade) were obtained from Merck kGaA (Darmstadt, Germany). Triethylamine and trifluoroacetic acid (analytical grade) were obtained from BDH Chemicals (Poole, England). Filter paper selected for this screening method was Whatman 31 ET Chr (cat. no: 3031–915) from Whatman International (Maidstone, UK). Extraction was carried out using a multimode (M-M) 100 mg, 1 ml (Biotage, VA, USA), and a C8 standard density disc SPE column, 1 ml (3M Empore, Bracknell, UK). Drug-free blood with heparin as anticoagulant was obtained from Mahidol University Hospital in Bangkok, Thailand.

### ▪ Preparation of samples

The difference in physicochemical properties between the drugs is a huge challenge when developing a filter paper method (Figure 1). First there are the hydrophilic drugs with basic properties (CQ, CQm and Q), or acidic properties (SDX). Second, moderately hydrophobic drugs with basic properties (MQ and Pyr). And third, highly lipophilic drugs with basic properties (LF), which require a high proportion of organic solvent to dissolve and a fairly high amount to stay in solution. It is important to take into account these different properties during all steps of the process.

Concentrated stock solutions of 1.00 mg/ml were prepared for each analyte (SDX 5.00 mg/ml). CQ, CQm and Q were dissolved in 0.1 M HCl. Pyr and MQ were dissolved in methanol:HCl (0.1 M) 50:50 v/v, SDX in 0.1 M NaOH and LF were dissolved in methanol:HAc 99.8:0.2 v/v. Stock solutions were stored in cryo tubes at +4°C. To simplify future dilutions, two sets of working solutions were prepared. Analytes dissolved in water solutions, such as CQ, CQm, Q and SDX, were added together in set 1 and any future dilutions were made with water. Analytes dissolved in methanol solutions, such as Pyr, MQ and LF, were added together in set 2 and any future dilutions were made with methanol:water 50:50 v/v. Working solutions were freshly prepared before use to create calibration standards and quality control (QC) samples.

Fresh whole blood was used to prepare calibration standards 50–3000 ng/ml (500–50,000 ng/ml for SDX). QC samples for CQm, CQ and Pyr were 250, 400 and 800 ng/ml, respectively; and for Q, MQ and LF 250, 600 and 2000 ng/ml, respectively. QC samples for SDX were 4000, 8000 and 20,000 ng/ml. The volume of working solution was less than 2% in whole blood. Spots of spiked blood (100 µl) were applied onto filter paper and kept at room temperature to dry. The DBS were kept at room temperature (∼21–24°C and relative humidity 35–60%) for 1 week (to let LF adsorb and stabilize) before transferring to ziplock bags and being stored at +4°C in a refrigerator until analyzed. Recovery samples were spiked in reconstitution solvent to simulate 100% recovery from spiked blood spot.

### ▪ Extraction procedure

The whole blood spot (100 µl) is cut out from the filter paper and then cut in three pieces and placed in a 2-ml microcup. In this extraction procedure, two sequential extractions from the same blood spot are carried out. In the first extraction, CQ, CQm, Q, SDX, Pyr and MQ are extracted by adding 1.5 ml methanol:HAc (0.5 M) 20:80 v/v; tubes are then placed on a sample tube rocker for approximately 1 h. Tubes are centrifuged at 7000 × g for 5 min and the liquid is then decanted into new 5-ml polypropylene tubes (keeping the paper in the microcups for further extraction) and 1 ml HAc (0.5 M) is added to the new tubes before transferring to SPE extraction with M-M column Table 1). For the second extraction, to extract LF, 1.5 ml acetonitrile:HAc (0.5 M) 50:50 v/v is added to each microcup and tubes are then placed on a sample tube rocker for approximately 1 h. Tubes are then centrifuged at 7000 × g for 5 min and the liquid is decanted into new 5-ml polypropylene tubes and 0.7 ml HAc (0.5 M) is added before transferring to SPE extraction with C8 disc column Table 1).

Eluates (in polypropylene tubes from M-M column and borosilicate glass tubes from C8 disc column) were evaporated under a gentle stream of air at 70°C. Dried samples from the M-M column were dissolved in 100 µl methanol:HCl (0.01 M) 10:90 v/v and 10 µl was injected into the LC system with the first described gradient program. Dried samples from the C8 disc column were dissolved in 100 µl methanol:HCl (0.01 M) 60:40 v/v and 10 µl was injected into the LC system with the gradient program described for LF.

### ▪ Instrumentation & chromatographic conditions

The LC system was a LaChrom Elite^®^ with two L2130 LC pumps, a L2200 injector set at 6°C, a L2300 column oven set at 25°C and a L2400 semi micro flow cell UV detector set at 280 nm (Hitachi, Tokyo, Japan). Data acquisition and quantification were performed using Hystar^TM^ and DataAnalysis^TM^ (Bruker, Bremen, Germany). The compounds were analyzed on a Phenomenex Gemini^®^ 5 µm C18 (150 mm × 2 mm) column and protected by a precolumn Security guard Gemini C18 (4 mm × 2 mm) (Phenomenex, Torrance, USA). The mobile phases were (A) acetonitrile:ammonium formiate (20 mM with 1 vol-% formic acid) (5:95 v/v) and (B) acetonitrile:ammonium formiate (10 mM with 1 vol-% formic acid) (80:20 v/v). Analytes extracted from the M-M SPE column were analyzed with this gradient mobile phase: initial conditions of A:B (93:7 v/v), with linear gradient up to A:B (10:90 v/v) over 7 min, held for 3 min then back to initial conditions in 1 min and then equilibrate for 4 min before next injection (total run time 15 min). LF extracted from the C8 SPE column were analyzed with this gradient mobile phase: initial conditions of A:B (60:40 v/v), with linear gradient up to 5 min A:B (7:93 v/v), held for 3 min then back to initial conditions in 1 min and then equilibrate for 4 min before next injection (total run time 13 min). Flow rate were 0.3 ml/min and injection volume 10 µl. The analytes were detected using an Esquire 4000 ion-trap mass spectrometer equipped with an ESI interface running in positive mode (Bruker Daltonics, Bremen, Germany). MS parameters were as follows: mass scan range *m/z* 200–800, nebulizer flow 40 psi (nitrogen), dry gas flow 9.0 l/min (nitrogen), dry temperature 365°C, ion spray voltage 5000 V and skimmer offset 38 V. The ion-trap will collect data of all compounds that are ionized in positive mode, have a *m/z* within the mass scan range and in detectable concentration. After the run, *m/z* for each analyte is then extracted from the data set.

### ▪ Validation

This screening assay is mainly developed for the identification of antimalarial drugs rather than to precisely quantify them. However, the validation process was carried out in a similar way as for a quantitative method to characterize variations in the method. Accuracy and precision of the method were estimated by analysis of 100-µl DBS in three replicates at three different concentrations over 5 days. Concentrations were determined using a nonweighted linear calibration curve analyzed at each run and within- and between-run precisions were calculated. The extraction recoveries were determined by comparing the precision samples (in triplicate) with a direct injected solution containing the same nominal concentration as the sample but prepared in reconstitution solvent, simulating a 100% extraction recovery.

Selectivity was evaluated by analysis of six blank samples from six different blood donors. Other antimalarial drugs were investigated for possible interference (e.g., amodiaquine, desethylamodiaquine, piperaquine, proguanil, cycloguanil, 4-chlorophenylbiguanide and halofantrine) by direct injection of pure standards into the LC system. Matrix effects were evaluated by blank samples from six different blood donors. The extracts were reconstituted with spiked reconstitution solvent and this solution also functioned as a pure reference sample. Concentration levels investigated were approximately the same as the lowest and highest QC levels. The peaks from the extract and reference sample were then compared to determine any matrix effects. Matrix effects were also evaluated by continuous post-column infusion. Blank samples from six different blood donors were processed and injected in the LC–MS and mass spectra were inspected for any visual matrix effects.

## Results & discussion

### ▪ Method development

Blood spots were allowed to dry for 7 days at room temperature before packed in ziplock bags and refrigerated at approximately 4°C. This was necessary for an accurate estimate of the detection limit for LF due to its time dependent absorption. LF has a continuous decrease in recovery from approximately 60% the first day to approximately 20% recovery after 4 to 7 days. This decrease has previously been prevented by pretreating the paper with 0.75 M tartaric acid [10], but this treatment has never been tested for the other drugs (e.g., for stability and recovery). This treatment also produces more interference in the chromatograms, especially for the more hydrophilic drugs with shorter retention times.

Earlier DBS methods have used both alkaline [11–14] or acidic solutions [15,16] for extraction of the different drugs and both solid-phase extraction and liquid–liquid extraction have been used as a clean-up step. A simple extraction step using pure acetonitrile or methanol with either direct injection or injection after evaporation/dissolution would be preferable. Extraction and direct injection was not possible since the paper itself absorbed approximately 200 to 300 µl of solvent. Approximately 400 µl of solvent would be required to enable direct injection, which resulted in too much dilution. An additional problem was severe band broadening for the early eluting hydrophilic drugs resulting in very broad and distorted peaks. An alternative was to evaporate the solvent and reconstitute it in an appropriate solvent for LC. However, this approach led to very low recovery for the lipophilic LF probably due to irreversible absorption to blood residues (i.e., proteins) during the evaporation step. Several of the drugs also showed low recovery due to the high (100%) organic content. The recovery could be increased if water was added to lower the amount of organic solvent, but this resulted in a more efficient extraction of endogenous blood components and increased interferences in the chromatogram.

Several organic solvents were evaluated but only methanol and acetonitrile yielded high recoveries. Acetonitrile was favorable for LF but not for the other drugs while the opposite was true for methanol – low recovery for LF while better for the others. The M-M SPE column could extract all drugs but also had some major drawbacks. The M-M column did not manage to purify the samples enough. Blood residues would adsorb some of the LF during the evaporation step giving random recoveries between 0 and 20%. LF also needed approximately 20–25% organic solvent in the load step to prevent precipitation, but this resulted in huge breakthrough on the SPE column for SDX with less than 2% recovery. This meant that it would be impossible to use a stronger SPE wash step.

The last possibility to extract all drugs was using a sequential extraction method. The aim was to extract all water-soluble drugs, leaving LF (and potentially other lipohilic drugs that could be of interest later) on the paper for a second extraction. This would hopefully improve extraction recoveries and produce cleaner extracts. All drugs except LF were optimized on the M-M column. Extraction with 20% methanol was the best choice, which also prevented LF from being co-extracted from the spots. Before loading the sample onto the SPE column it had to be diluted to 10% methanol to avoid breakthrough of SDX and elution was accomplished using methanol:triethylamine 97:3. Another advantage with separating LF from the others was that the reconstitution solvent for the other drugs could be limited to only 10% methanol, thereby avoiding band broadening when injected into the LC system. LF was then extracted from the blood spot using a combination of acetonitrile:acetic acid 0.5 M 50:50 [10] and applied onto a C8 disc SPE column. This produced a cleaner extract and avoided problems of adsorption during the evaporation.

### ▪ Validation

A huge advantage of the ion-trap compared with a single or triple quadrupole detector for these applications is that the ion-trap will collect data for all compounds having a *m/z* ratio within the mass scan range provided that they are ionized. Once the total ion chromatogram data has been acquired, *m/z* chromatograms for each relevant analyte are extracted. This means that it is possible to later go back into the data set and look for other drugs that might be in the samples. The main disadvantage of the ion-trap for quantitative measurements is that the linear range is somewhat smaller than for a quadrupole instrument. Three internal standards were added during the first validation series, one with properties suitable for the highly hydrophilic drugs (i.e., CQm, CQ and Q), one internal standard suitable for SDX and one with lipophilic properties for LF. After evaluation of validation data it was concluded that internal standards had no major effect in correcting deviations in the method. To enable the assay the possibility to also capture other drugs, the internal standards were omitted (i.e., the chromatograms would get less crowded). Precision and accuracy were calculated for the different QC levels shown in Table 2.

Within-day precision (n = 15) was below 20% for all drugs and concentrations except the lowest QC (250 ng/ml) for Pyr that deviated slightly more. Between-day precision (n = 5) was also 20% or below. Accuracy was within 20% for all drugs and concentrations except the lowest QC for SDX and the lowest QC for LF which both deviated more. The large deviation in accuracy at the lower concentration region for LF is probably due to the use of a sequential extraction. Although LF is not soluble in water, very small amounts will probably solvate and this would affect the accuracy of the lowest concentrations the most. The lowest QC for LF is also very close to its detection limit (200 ng/ml). On the other hand, drug levels of SDX in blood are very high and would be in the upper region of the calibration range or above making it easy to identify their presence.

The total extraction recoveries were in the range of 60 to 75% for CQ, CQm and Pyr; 75 to 85% for Q; 25 to 35% for SDX; 40 to 50% for MQ and approximately 20% for LF. Low recovery for SDX is not a problem due to a high biological concentration in blood. LF, however, could have been higher but 20% is about the best recovery on a filter paper that has not been pretreated with tartaric acid and should be enough to detect LF at least 7 days after administration.

Earlier published papers present recoveries of 70 to 90% for CQ and 90% or above for CQm [11,12]; for Pyr approximately 35–45% [13] or 58–67% [15] and for Q approximately 79–103% [17]. Earlier reported recoveries for SDX is 66–77% [16] or 58–67% [15] and for MQ approximately 70–80% [14]. There are no published data for LF, but with pretreated filter paper recovery of 60% [10] is reported or 45–50% [18] if blood is pretreated before applying to the paper.

Selectivity was evaluated from six blank blood samples from six different blood donors and no endogenous compound peaks were identified. Interferences from other antimalarial drugs were also investigated and the retention order on the Gemini C18 column is as follows (with at least 0.1 min between peak apexes): piperaquine, CQm, desethylamodiquine, CQ, amodiaquine, Q, 4-chlorophenylbiguanide, cycloguanil, Pyr, proguanil, SDX, MQ, halofantrine and LF. An overlay of the drugs in this assay is shown in Figure 2, extracted from a blood spot (250 ng/ml) with M-M column. Figure 3 shows the corresponding LF extraction with a C8 disc column. Matrix effects were evaluated by comparing the peaks from extracted blank samples spiked with reference sample (spiked reconstitution solvent) and compared with the same pure reference solution at two different concentrations. No major matrix effects were observed form the six different donors and any variations were within that of the method itself. Post-column infusion was also performed. There were no visible matrix effects for any of the drugs from any of the blank extracts from the six blood donors.

No stability study was performed in this paper. Earlier reports suggest that CQ and CQm are stable in DBS for at least 7–12 weeks at 20°C [19,20], Q for at least 2 months at 37°C [17], and Pyr for several months at 19–22°C and for at least 2 weeks at 35°C [21]. SDX was found to be stable for at least 40 days at 37°C and -20°C for at least 4 years [16]. Less than 10% decrease after 50 days storage at 37°C is reported for MQ [14]. Stability studies of LF have shown good stability at 22 and 37°C for at least 3 months in both treated (0.75 M tartaric acid) and untreated papers [10].

## Conclusion & future perspective

This screening assay may be a useful tool to improve the accuracy and validity of future investigations of drug use. With detection limits of 50 ng/ml and a relatively long half-life for most of the drugs in this assay, it is possible to detect drugs up to a few weeks after drug intake. The use of ion-trap MS with an ESI interface and total ion chromatograms make it possible to extract and identify other common antimalarials and other drugs that might be of interest at a later stage. A very simple extraction method would be optimal but with the huge differences in physicochemical properties it was not possible to achieve this. LF with a detection limit of 200 ng/ml is detectible approximately 1 week after drug treatment and at that drug level it is possible to use LC with UV detection with approximately the same detection limit. However, the possibility to later go back to extract and identify *m/z* of an unidentified peak as in ion-trap LC–MS is not possible. In the future, it would be desirable to develop a faster and simpler sample preparation technique if possible. Preferably a fast and easy extraction such as extracting the blood spot with solvent, adding internal standard then mixing, centrifuging and carrying out direct analysis with HPLC–MS/MS. The possibility of using direct desorption techniques for DBS is interesting [22–25], but these techniques will likely be vulnerable to matrix effects due to the lack of proper sample clean-up. Future development of better extraction techniques, more efficient separations and more sensitive mass spectrometers will hopefully pave the way for sensitive high-throughput screening methods.

**Table 1.** Solid-phase extraction procedure.

**SPE step**	**Samples from first extraction, solvents for multimode column**	**Volume (ml)**	**Samples from second extraction, solvents for C8 column**	**Volume (ml)**
Activation	Methanol	1.0	Methanol	0.5

Conditioning	Acetic acid (0.5 M)	1.0	Acetonitrile:water:acetic acid (30:69.5:0.5 v/v)	0.3

Loading	Sample	2.3	Sample	2.0

Washing	Acetic acid (0.5 M)	1.0	Acetonitrile:water:acetic acid (30:69.5:0.5 v/v)	0.5

Elution	Methanol:triethylamine (97:3 v/v)	1.0	Methanol:trifluoroacetic acid (99.9:0.1 v/v)	1.0

*Flow rate is 1 ml/min or less during load and elution step.*

**Table 2.** Accuracy and precision for the drugs on Whatman 31 ET Chr filter paper dried blood spots.

**Analyte**	**Added (ng/ml)**	**Found (ng/ml)**	**Within-day (n = 15) relative standard deviation (%)**	**Between-day (n = 5) relative standard deviation (%)**	**% deviation found versus added**
CQm	250	288	14	4.5	15

	400	413	7	13	3.5

	800	847	5	15	6

CQ	250	276	10	7	10.5

	400	424	12	5	6

	800	894	9.5	3.5	12

Quinine	250	279	11.5	6.5	11.5

	600	660	10	4	10

	2000	2151	5.5	2	7.5

Pyr	250	268	22	20	7.5

	400	384	9.5	13	-4.5

	800	775	11.5	5.5	-3.5

SDX	4000	6260	9.5	3.5	56

	8000	8100	11	8	1.5

	20000	21310	4	10	7

MQ	250	282	11.5	16	13

	600	659	9	8	10

	2000	2077	4.5	4.0	4

LF	250	347	15	18	39

	600	522	13.5	14	-13

	2000	2140	11.5	6	7

*CQ: Chloroquine; CQm: Desethylchloroquine; LF: Lumefantrine; MQ: Mefloquine; Pyr: Pyrimethamine; Q: Quinine; SDX: Sulfadoxine.*

**Antimalarial**

Any drug used for treatment of malaria.

**Desethylchloroquine**

Metabolite of chloroquine.

**Whatman 31 ET Chr**

A 0.5 mm thick filter paper with fairly soft surface that will absorb blood fast to a well-defined blood spot.

**Ion-trap**

Ions are confined between electrodes and a mass spectrum is obtained by increasing the radiofrequency amplitude, which will destabilize the ions in ascending mass-to-charge ratio (*m/z*) and eject them towards the detector. All information in the selected mass range is saved and *m/z* of chosen drugs is extracted by computer software. Ion-traps have a limited dynamic range and are not so suitable for quantification.

**Solid-phase extraction**

A sample preparation technique to get rid of impurities that might interfere in sample detection.

**Executive summary**

- ▪ This screening method can be used for determining drug use in areas where self-medication is common.
- ▪ The method is able to detect and identify seven of the most common antimalarials.
- ▪ A sequential extraction efficiently and successfully extracts several drugs with huge differences in their physicochemical properties. Using an ion-trap mass spectrometer and total ion chromatograms enables the possibility of post-acquisition querying of the dataset for other common antimalarials that might be present in the blood spots.
- ▪ With detection limits of 50 ng/ml and relatively long half-lives for most of the drugs in this assay, it is possible to detect drugs up to a few weeks after drug intake.
